# *Treponema pallidum* induces pathological injury of the rabbit testis and sperm through NLRP3 inflammasome activation-mediated pyroptosis

**DOI:** 10.1186/s12865-026-00835-7

**Published:** 2026-05-09

**Authors:** Tong Zheng, Meiping Ye, Yilan Yang, Linxin Yao, Danyang Zou, Chunjie Liao, Xin Feng, Tingli Tian, Zhuojun Tang, Pingyu Zhou

**Affiliations:** 1 STD Institute, Shanghai Skin Disease Hospital, School of Medicine, Tongji University, Shanghai, China; 2 Department of Dermatology, Xinhua Hospital, Shanghai Jiaotong University School of Medicine, Shanghai, China

**Keywords:** *Treponema pallidum*, Testis, Sperm, NLRP3 inflammasome, Pyroptosis

## Abstract

**Background:**

*Treponema pallidum* (*T. pallidum*) can invade various organs and tissues. However, there have been few studies on the pathological injury of *T. pallidum* to the testis and sperm. The NOD-like receptor protein 3 (NLRP3) inflammasome, upon activation, leads to pyroptosis—a programmed, pro-inflammatory cell death process, and promotes inflammatory responses. Previous investigations have found that the NLRP3 inflammasome is elevated in the testis tissues of rabbits infected with *T. pallidum*. This study aimed to investigate whether *T. pallidum* induced pathological injury of the rabbit testis and sperm through NLRP3 inflammasome-mediated pyroptosis.

**Methods:**

While rabbits in the *T. pallidum* group received an intratesticular injection of 1 mL of a bacterial suspension containing 10⁷/mL treponemes, those in the sham group were administered an equal volume of normal saline to serve as the injection procedure control. 14 days later, testis tissues were harvested for IHC and H&E staining, and analysis of qPCR. In vitro, GC-2spd cells were stimulated by *T. pallidum* (MOI = 20). After administering MCC950, the expression levels of the inflammasome NLRP3 and downstream pyroptosis-related molecules were subsequently detected.

**Results:**

*T. pallidum* caused obvious pathological damage to the rabbit testis and sperm reduction. Meanwhile, the gene expression levels of NLRP3, ASC, GSDMD, Caspase-1, IL-Iβ, and IL-18 in the *T. pallidum* group were higher than those of the sham group. Additionally, *T. pallidum* induced cell injury and pyroptosis in GC-2spd cells, which was improved by the NLRP3 inflammasome inhibitor MCC950.

**Conclusions:**

Our findings validated that *T. pallidum* induced pathological damage to the testis and sperm reduction via NLRP3 inflammasome activation and pyroptosis. By specifically inhibiting the NLRP3 inflammasome, MCC950 has the potential to ameliorate sperm cell damage under in vitro conditions via the blockade of pyroptotic cell death.

**Supplementary Information:**

The online version contains supplementary material available at 10.1186/s12865-026-00835-7.

## Background

Syphilis remains a common global health issue. It is a chronic, systemic sexually transmitted infection whose causative agent is the bacterium *Treponema pallidum* [1, 2]. *Treponema pallidum* can invade different organs and tissues of the human body [3, 4]. In advanced stages, syphilis can cause orchitis [5], which can lead to severe injury to the testicles. However, there are few studies that focus on the pathological damage of the human testis and the effect of *Treponema pallidum* on sperm.

The NLRP3 inflammasome, a critical intracellular multiprotein complex, functions as a central regulator of inflammatory responses in numerous pathological conditions. Upon detection of microbial or danger-associated signals, its activation nucleates the assembly of a large speck-like complex containing ASC, which in turn activates Caspase-1. The activated Caspase-1 then proteolytically cleaves gasdermin D (GSDMD), generating an N-terminal fragment (N-GSDMD). This fragment translocates to the cell membrane and oligomerizes to form permeable pores. The formation of these pores, coupled with the secretion of the proinflammatory cytokines interleukin-1β (IL-1β) and interleukin-18 (IL-18), ultimately drives the execution of pyroptosis—a form of programmed cell death [6–8].

Rabbits serve as a common and readily available model for syphilis, as *T. pallidum* infection induces immune-mediated pathology mimicking human disease [9]. Notably, testicular levels of NLRP3 were elevated in infected rabbits, reaching a maximum at day 14 [10]. Based on these findings, we therefore propose that *T. pallidum*-mediated activation of the NLRP3 inflammasome and the subsequent pyroptosis constitute a key mechanism underlying testicular damage. In this study, we investigate NLRP3 inflammasome-mediated pyroptosis in vivo and in vitro, and investigate whether the NLRP3 inflammasome inhibitor MCC950 reduces pyroptotic death of spermatogenic cells (GC-2spd).

## Methods

### *T. pallidum Nichols* strain

*T. pallidum Nichols* strain was gifted by the Syphilis research team of Washington University and transferred by the Clinical Laboratory of Zhongshan Hospital Affiliated to Xiamen University.

### Animal experiments

Male New Zealand White rabbits were purchased from Leagene Biotechnology Co., Ltd. (Beijing, China). Rabbits (3 months old, 2.5–3.0 kg) were inoculated intratesticularly with the *T. pallidum* Nichols strain, following an established protocol [9]. All animals were individually housed under controlled temperatures (16–18 °C) and provided with an antibiotic-free diet and water ad libitum. Prior to the experiment, each rabbit was confirmed seronegative by both treponemal and non-treponemal tests. For the infection group, a 1 mL bacterial suspension containing 10⁷ *T. pallidum* per mL was administered into each testis, whereas the sham group received an equivalent volume of sterile normal saline. The animals in the two groups were euthanasia at 14 days post-infection, and blood and testes were then harvested for the following experimental analysis. Meanwhile, lipopolysaccharide (LPS) (300 µg/kg) was injected into the testicles of rabbits as the positive control group. Rabbits were euthanized by intravenous injection of an overdose of sodium pentobarbital (100 mg/kg, 60 mg/mL solution) via the marginal ear vein. The injection was performed smoothly and rapidly (completed within 5–10 s). The animals were conscious before injection, but lost consciousness within seconds after the start of injection, followed by cardiac and respiratory arrest. Death was confirmed by absence of heartbeat and breathing for at least 5 min. This euthanasia method is recommended by the AVMA Guidelines for the Euthanasia of Animals (2020 Edition) as a humane and rapid technique for rabbits. The high dose (≥ 100 mg/kg) ensures rapid loss of consciousness and minimal distress, and it does not interfere with subsequent histopathological evaluation of testicular and sperm tissues.

### Serology test

Following centrifugation of the collected rabbit blood at 3000 rpm for 10 min, the obtained serum was serially diluted. The TPPA (*Treponema pallidum* particle agglutination assay, Fujirebio, Japan) and TRUST (Toluidine Red Unheated Serum Test, Rongsheng, China) titers were then quantitatively determined in the diluted sera using the corresponding commercial kits.

### Immunohistochemistry (IHC) staining

Testicular tissues were collected and processed for immunohistochemical (IHC) analysis by fixation in 10% neutral-buffered formalin and subsequent paraffin embedding. For histological assessment, tissue sections were incubated with a primary antibody specific to *Treponema pallidum* (JIHAO BIOTECHNOLOGY, China) and then examined under a light microscope (Olympus, Japan).

### Hematoxylin and Eosin (H&E) staining

Testicular tissue samples collected from all experimental groups were prepared for histological evaluation using hematoxylin and eosin (H&E) staining. In brief, tissues were fixed in 10% neutral-buffered formalin for 24 h, dehydrated in an ascending ethanol gradient, embedded in paraffin, and sectioned into thin slices. The sections were dried and stained to visualize morphological features. Histopathological changes in testicular structure were examined under a light microscope (Olympus, Japan) for qualitative assessment.

### Cell culture and experimental design

Mouse spermatogenic cells (GC-2spd) (Boster Biotech) were cultured in DMEM (Gibco, USA) medium, which consists of 10% fetal bovine serum (FBS) (Gibco, USA) and 1% penicillin/streptomycin, in an atmosphere of 90% air and 10% CO_2_ at 37 °C. Following trypsinization with 0.05% trypsin/1 mM EDTA (Gibco, USA) at 80–90% cell density, the cultures were plated in 6-well plates (10⁵ cells/well) and randomly allocated to three groups: [1] Control (Ctrl) (PBS); [2] *T. pallidum* (TP)(MOI = 20); [3] TP+MCC950(5 *µ*M). All cells were then incubated for 24 h in medium without 1% penicillin/streptomycin.

### Cell viability assay

A Cell Counting Kit-8 (CCK-8; SuperKine™, Abbkine, China) was employed to determine cell viability according to the manufacturer’s instructions. GC-2spd cells were cultured in 96-well plates and subjected to the designated treatments. After treatment, 10 µL of CCK-8 solution was added to each well, and the plates were incubated for 1–4 h under light-protected conditions. Absorbance was measured at a wavelength of 450 nm using a microplate reader. The viability of cells in each group was expressed as a percentage and calculated using the formula: cell viability (%) = (OD_sample_- OD_blank_) / (OD_control_- OD_blank_) × 100%.

### Caspase-1 activity assay

The activity of caspase-1 was assessed with a specific detection kit (Elabscience, China) as per the manufacturer’s guidelines. After measuring the absorbance at a wavelength of 405 nm, the corresponding activity levels across groups were computed using the established calculation method: (OD_sample_- OD_blank_) / (OD_control_- OD_blank_) × 100%.

LDH activity assay

### LDH activity assay

Cytotoxicity resulting from pyroptotic cell death was assessed by measuring lactate dehydrogenase (LDH) activity in the cell culture medium. This measurement was performed with an LDH assay kit (Beyotime, China), following the supplier’s recommended instructions. The results were compared with the maximal LDH released from cells.

### Calcein AM/Propidium Iodide (PI) staining assay

According to the manufacturer’s instructions (Elabscience, China), live and dead cells were quantified with a Calcein AM/PI double staining kit. After experimental interventions, cells were incubated with the staining solution for 30 min at 37 °C. In this assay, metabolically active live cells esterify non-fluorescent Calcein AM to intensely green-fluorescent calcein, whereas PI labels deceased cells by binding to DNA and producing red emission. All samples were visualized under a fluorescence microscope (Zeiss, Germany).

RNA isolation and quantitative Real-Time PCR (qRT-PCR)

### RNA isolation and quantitative Real-Time PCR (qRT-PCR)

Total RNA was isolated from rabbit testicular tissues and GC-2spd cells. The RNA concentration was determined using a NanoPhotometer^®^ N50 (Implen, Germany). Following this, cDNA was synthesized from the extracted RNA with a high-capacity cDNA reverse transcription kit (Takara, Japan). Finally, quantitative real-time PCR (qRT-PCR) was conducted on a CFX Connect™ Real-Time System (BIO-RAD, USA) following optimized thermal cycling conditions. The relative mRNA expression levels were analyzed via the 2^−ΔΔCT^ method, using GAPDH mRNA as the endogenous reference for normalization [10]. The following primer pairs were used: NLRP3, (5’-GTTCTAATAAGGGCGAGGCG-3′ and 5’-ACAGTTCCGTGGTCACTTCT-3′); ASC, (5’-GGGCGGGATACAAAGGGGTA-3′ and 5’-CTCCCTCCACCCCGCTAATC-3′); Caspase-1, (5’-GTACCTGTGCATCGACCTGT-3′ and 5’-ATCGTCGAAGTCGAAGCAGG-3′); GSDMD, (5’-GGACTCTTTCCCTAGGTGTGG-3′ and 5’-AAAGCACTTCCCACCTTCCT-3′); IL-1β, (5’-GGCACAACAGATCGCTTTGG-3′ and 5’-TCTCACTGGTGAGCTCAGGT-3′); IL-18, (5’-GGCACAACAGATCGCTTTGG-3′ and 5’-GGGCAGCAGAACGTGAAATC-3′); and GAPDH, (5’-GTTTGTGATGGGCGTGAACC-3′ and 5’-GTCATGAGCCCCTCCACAAT-3′). NLRP3, (5’-ATTACCCGCCCGAGAAAGG-3′ and 5’-TCGCAGCAAAGATCCACACAG-3′); ASC, (5’-CTTGTCAGGGGATGAACTCAAAA-3′ and 5’-GCCATACGACTCCAGATAGTAGC-3′); Caspase-1, (5’-ACAAGGCACGGGACCTATG-3′ and 5’-TCCCAGTCAGTCCTGGAAATG-3′); GSDMD, (5’-CCATCGGCCTTTGAGAAAGTG-3′ and 5’-ACACATGAATAACGGGGTTTCC-3′); IL-1β, (5’-GCAACTGTTCCTGAACTCAACT-3′ and 5’-ATCTTTTGGGGTCCGTCAACT-3′); IL-18, (5’-GACTCTTGCGTCAACTTCAAGG-3′ and 5’-CAGGCTGTCTTTTGTCAACGA-3′); and GAPDH, (5’-TGCACCACCAACTGCTTAGC-3′ and 5’-GGCATGGACTGTGGTCATGAG-3′).

### Western blot analysis

Western blot analysis was performed as described previously [11] using antibodies against NLRP3 (1 : 1000, Abcam, ab263899), ASC (1 : 500, Santa Cruz, sc-271054), c-caspase-1 (1 :500, Novus, NBP1-76605), IL-1*β* (1 : 1000, Abcam, ab254360), IL-18 (1 : 1000, Abcam, ab207323) and GAPDH (1 : 2000, Beyotime, AF1186). GAPDH served as an internal control. The protein bands were detected with the GelDoc XR System (BIO-RAD, USA), and protein densitometry was analyzed by ImageJ software.

### Statistical analysis

All data are presented as the mean ± SD and were analyzed using SPSS 25.0 (IBM Corp.). Statistical comparison of the means between two groups was accomplished by the Student’s t-test (parametric) or the Mann-Whitney U test (nonparametric). Statistical comparisons among multiple groups were performed using one-way analysis of variance (ANOVA), followed by post-hoc tests with Bonferroni adjustment for multiple comparisons. The significance threshold was set at *P* < 0.05. All statistical analyses and graphing were conducted using GraphPad Prism software (version 9.0).

## Results

### *T. pallidum* induced markedly pathological damage to the testis of rabbits

**Fig. 1:**
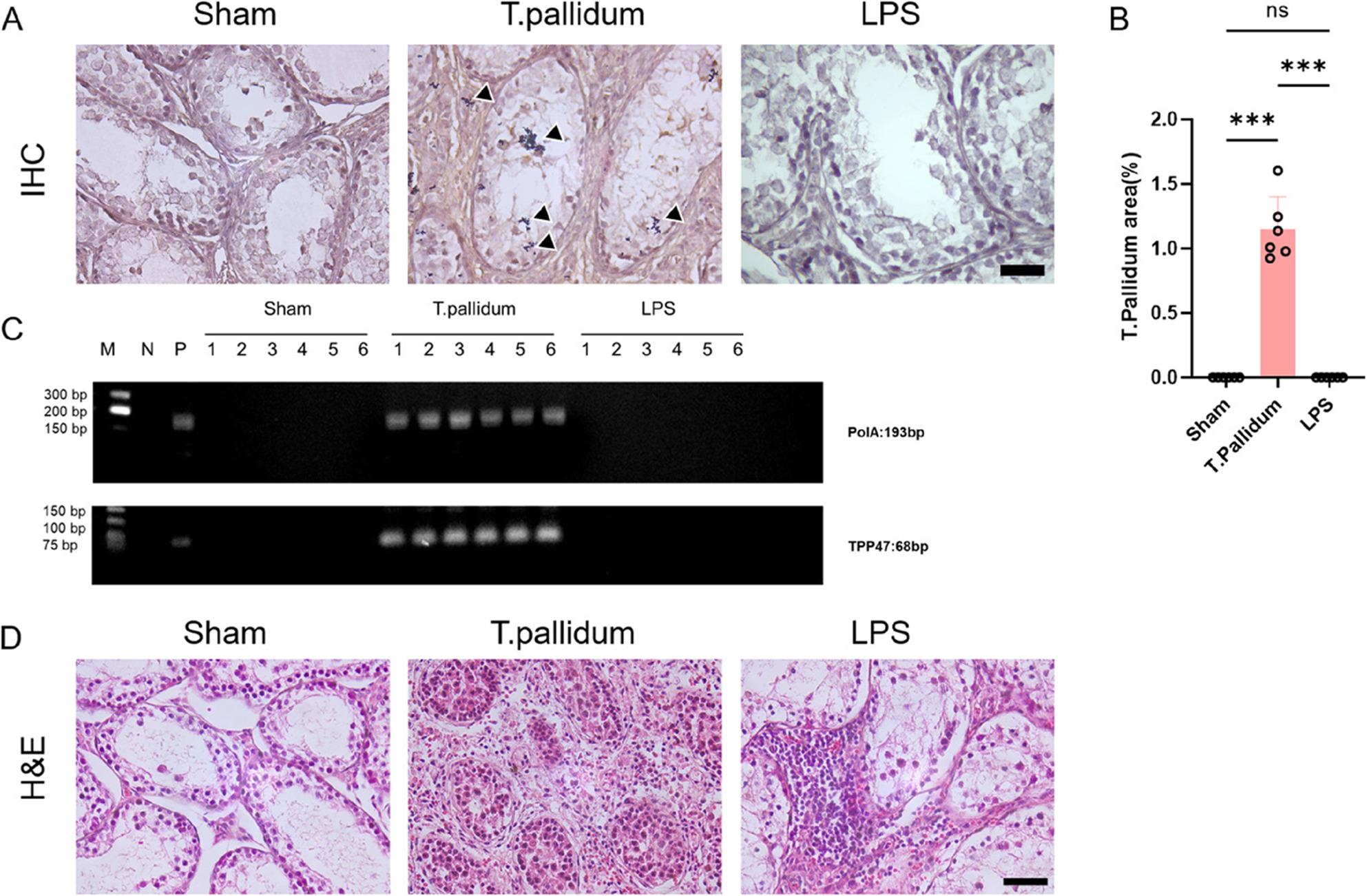
*T. pallidum* invaded the testes of rabbits and induced injury. **A**, **B** Representative IHC staining of paraffin sections and *T. pallidum* area (%) of the testicular tissues from rabbits. Scale bar = 50 μm. n = 6 per group. **C** Specificity of the polA and TPP47 assay detected by electrophoresis. Lane M, 500 bp marker; lane N, negative control; lane P, positive control; lane sham (1-6), sham group; lane *T. Pallidum* (1-6), *T. Pallidum* group; lane LPS (1-6), LPS group. **D** Representative H&E staining of paraffin sections of the testicular tissues from rabbits. Scale bar = 50 μm. n = 6 per group

Compared with that before inoculation, the swelling of the rabbit testis was most obvious 14 days after *T. pallidum Nichols* strain inoculation and could not be retracted. As shown in Table 1, serum TPPA and TRUST were positive in rabbits 14 days after inoculation with *T. pallidum*, and the weight of the testis increased markedly. According to previous studies, the normal rabbit testis is composed of closely spaced seminiferous tubules and interstitial areas. Seminiferous tubules consist of well-arranged spermatogonia, and mature spermatozoa are usually found in the lumen. The intertubular spaces or interstitial areas contained venules, arterioles, and interstitial cells, and a small number of scattered lymphocytes were also seen [12]. The IHC staining of rabbit testes showed that *Treponema pallidum* was located in the seminiferous tubules and interstitial area in the *T. pallidum* group (Fig. 1A and B), and PCR also suggested that the *T. pallidum* group presented specific bands for *PolA* and *TPP47*, which are specific genes of *Treponema pallidum* (Fig. 1C). In the *T. pallidum* group, mononuclear cells, lymphocytes, and macrophages were infiltrated in the interstitial and vascular areas of the testis; the interstitial fibrosis was observed, the seminiferous tubules were significantly atrophied, and the number of mature sperm was evidently reduced (Fig. 1D). These results indicated that *T. pallidum* can cause markedly pathological damage to the rabbit testis.

**Table 1:** Characteristics of the sham and *T.pallidum* group after 14 days

	sham	T.pallidum
Serum TPPA	(-)	(+)
Serum TRUST	0	≥ 1:1024
Testicle weight (g)	2.24 ± 0.16	7.94 ± 0.29 ***
Body weight (kg)	2.85 ± 0.16	2.88 ± 0.14

Data are expressed as the mean ± SD. *n* = 6. ***P < 0.001 versus sham group

### The testicular injury and sperm reduction of rabbits were associated with increased activation of the NLRP3 Inflammasome

**Fig. 2:**
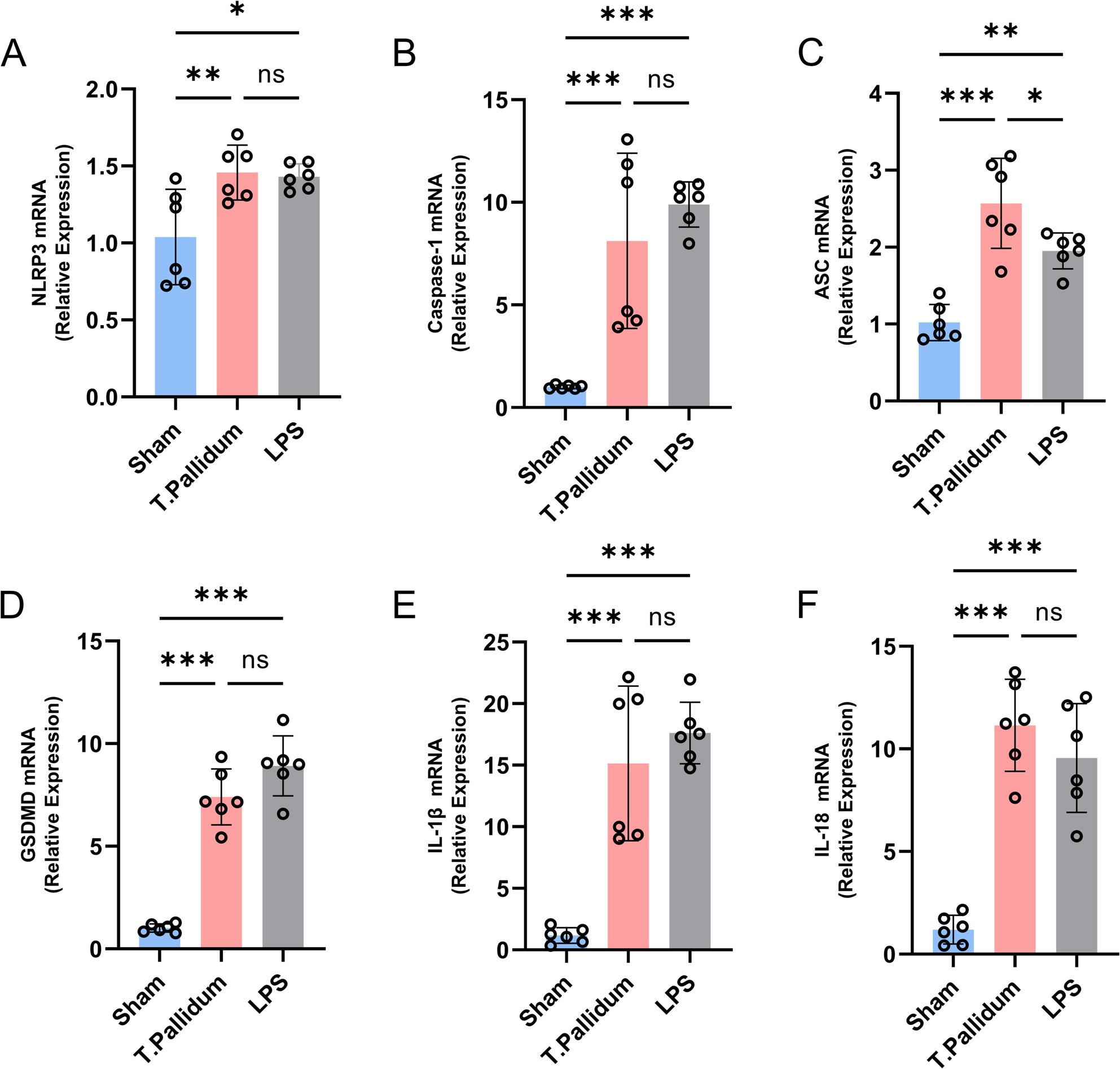
NLRP3 inflammasome and its downstream pyroptosis molecules increased in the rabbit testicles invaded by *T. pallidum*. **A**-**F** The mRNA expression levels of NLRP3, ASC, Caspase-1, GSDMD, IL-1β, and IL-18 in rabbit testicular tissues of each group were evaluated by RT-PCR, respectively. *n* = 6 per group. Data are means ± SD. *P < 0.05, **P < 0.01, and ***P < 0.001

Prior evidence indicates an upregulation of NLRP3 inflammasome expression in the testes of *T. pallidum*-infected rabbits [10], and its activation is known to induce germ cell pyroptosis [13]. Given this background, we investigated the testicular expression of key pyroptosis-related molecules (NLRP3, ASC, GSDMD, Caspase-1, IL-1β, IL-18) following *T. pallidum* challenge. Consistent with these findings, our qRT-PCR results (Fig. 2) revealed that infection significantly upregulated the mRNA levels of all these components compared to the sham group. Notably, this increase was comparable to the positive control LPS group. These results indicated that the increased expression of NLRP3 and downstream pyroptosis molecules is involved in the process of testicular damage and sperm reduction.

### *T. pallidum* induced cell injury and pyroptosis in cultured GC-2spd cells, which was attenuated by the NLRP3 inflammasome inhibitor

**Fig. 3:**
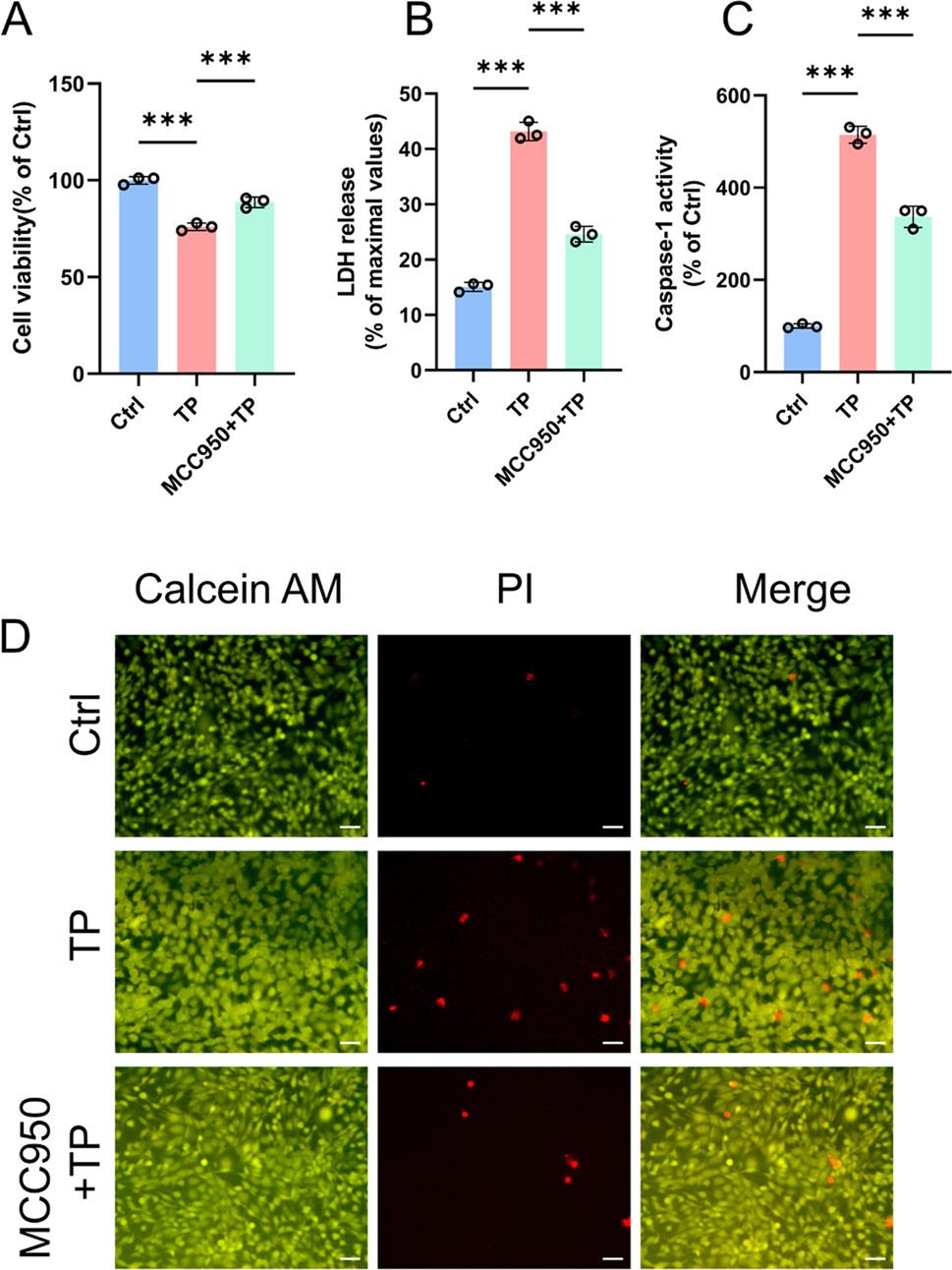
MCC950 reduced the cell pyroptotic death induced by *T. pallidum* in vitro. **A**-**C** The cell viability, the LDH release, and the caspase-1activity were assessed. **D** Calcein AM/PI was used to detect pyroptosis. Data are expressed as the mean ± SD. *n* = 3 per group. *P < 0.05, **P < 0.01, and ***P < 0.001

Compared with the Ctrl group, *T. pallidum* stimulation significantly decreased the cell viability (Fig. 3A), while increasing the LDH release (Fig. 3B) in cultured GC-2spd cells. In addition, we subsequently evaluated pyroptosis by measuring caspase-1 activity and by performing Calcein-AM/PI double staining, which serves as a definitive method for monitoring cell death and specifically identifying pyroptosis [14]. As shown in Fig.3C, D and T. *pallidum* notably induced caspase-1 activity and pyroptotic cell death compared with the Ctrl group. However, when the NLRP3 inflammasome inhibitor MCC950 was used, the cell viability (Fig. 3A) was significantly increased and reversed the cell injury induced by *T. pallidum*. In addition, the increased LDH level (Fig. 3B) and caspase-1 activity level (Fig. 3C) were significantly reversed by MCC950. Meanwhile, treatment with MCC950 notably suppressed the pyroptotic cell death (Fig. 3D). These results indicated that *T. pallidum* can induce pyroptosis at the cellular level.

### The NLRP3 inflammasome inhibitor downgraded NLRP3 inflammasome activation and the expression of executors of pyroptosis in cultured GC-2spd cells exposed to *T. pallidum*

**Fig. 4:**
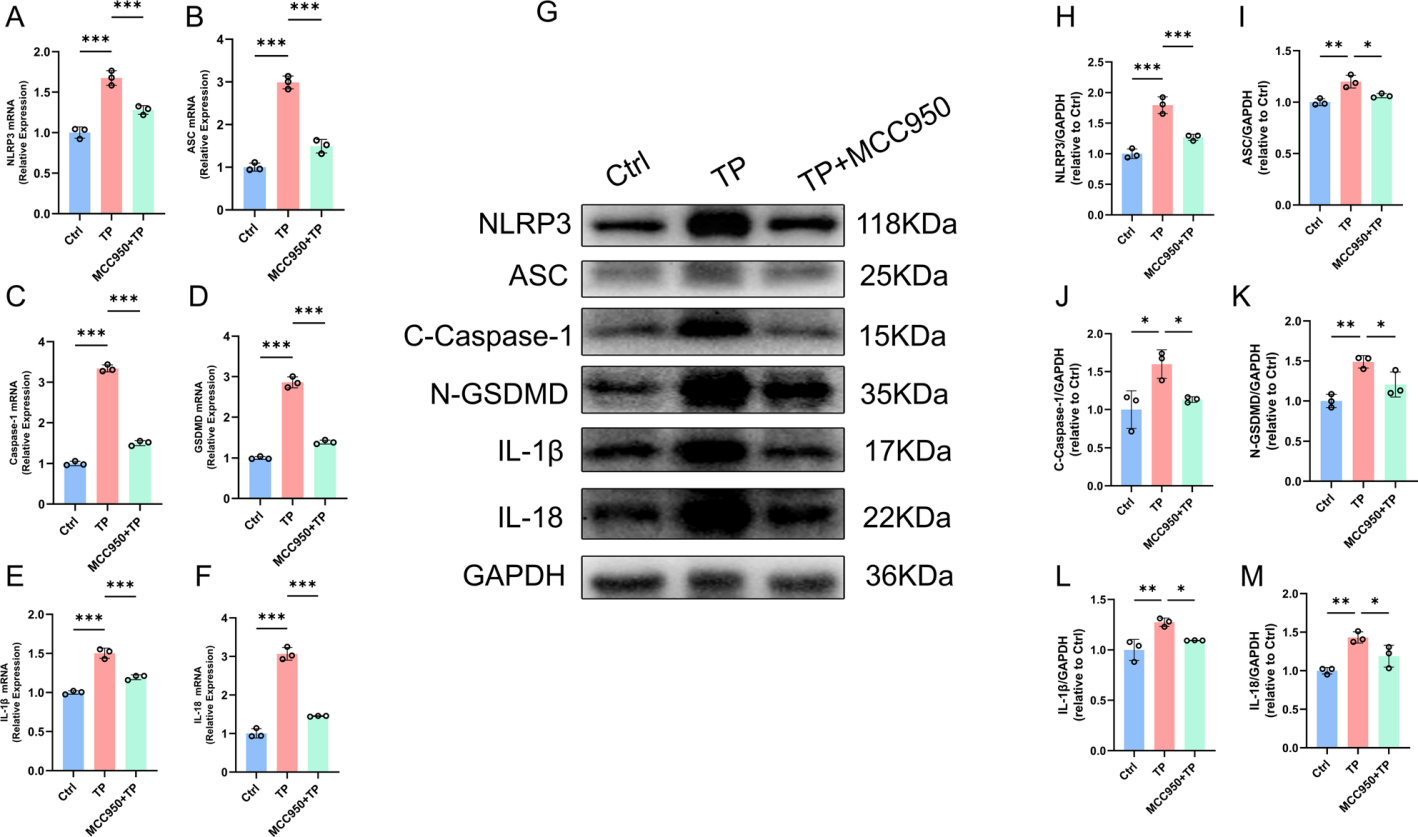
MCC950 reduced the expression levels of NLRP3 and executors of pyroptosis. **A**-**F** The mRNA expression levels of NLRP3, ASC, Caspase-1, GSDMD, IL-1β, and IL-18 in GC-2spdc ells of each group were evaluated by RT-PCR. **G**-**M** Protein expression levels of NLRP3, ASC, C-Caspase-1, N-GSDMD, IL-1β, and IL-18 by TP in GC-2spd cells of each group were evaluated by Western Blot. Data are expressed as the mean ± SD. *n* = 3 per group. *P < 0.05, **P < 0.01, and ***P < 0.001

To investigate the involvement of the NLRP3 inflammasome and pyroptosis in cellular responses to *T. pallidum*, we quantified the expression of key molecules at both the mRNA and protein levels. The results indicated that *T. pallidum* stimulation remarkably upregulated the transcript levels of NLRP3, ASC, Caspase-1, GSDMD, IL-1β, and IL-18 (Fig. 4A-F). Consistently, western blot analysis confirmed a corresponding elevation in the protein abundance of NLRP3, ASC, cleaved Caspase-1, N-terminal GSDMD, IL-1β, and IL-18 (Fig. 4G-M). Notably, the NLRP3-specific inhibitor MCC950 effectively suppressed the activation of this signaling axis and the expression of all these pyroptosis-related molecules (Fig. 4A-M), indicating a coordinated transcriptional and translational regulation of this pathway. The synchronous upregulation at the mRNA level reveled that the activation of the NLRP3 inflammasome was not solely dependent on post-translational modifications, but began at a more upstream stage - gene transcription. This transcriptional enhancement provided the necessary foundation for the assembly of the inflammasome and the explosive execution of its functions. The Western Blot results confirmed that the increase in transcription was efficiently converted into the accumulation of functional proteins. The significant increase in mature C-Caspase-1 and N-GSDMD fragments is direct evidence of the successful assembly of the inflammasome and its ability to cleave substrates. At the same time, the increase in mature IL-1β and IL-18 levels indicated that the final inflammatory effect of the pyroptosis process has been successfully released.

## Discussion

Syphilis is a systemic disease caused by *Treponema pallidum*, the pathogen of syphilis, which can infect various organs and tissues of the human body, such as the skin, mucosa, eye, nervous system, and internal organs [1, 3, 4]. Cases of syphilitic orchitis and syphilitic epididymo-orchitis have been reported [5, 15–18]. One previous study showed that *Treponema pallidum* was detected in the semen of a patient with syphilis [19]. Therefore, the impact of *Treponema pallidum* on the testis has attracted our focus, and whether it can cause male fertility problems by affecting the reproductive system and sperm should be paid enough attention. Since the rabbit model of syphilis can imitate many of the clinical symptoms of human syphilis infection [9], we chose the rabbit model of syphilis to study the pathological injury of the testis and sperm in vivo. In the present study, we found obvious testicular swelling and non-retraction at 14 days after *T. pallidum* injection. TPPA and TRUST were used to determine the success of the rabbit model of syphilis. *Treponema pallidum* can invade the seminiferous tubules and interstitial area and result in marked pathological injury to the rabbit testis and sperm death. In vitro, *T. pallidum* induced pyroptotic cell injury in cultured GC-2spd cells, which was alleviated by the NLRP3 inflammasome inhibitor MCC950. Our results revealed that both the NLRP3 inflammasome activation and executors of pyroptosis, including ASC, Caspase-1, GSDMD, IL-1β, and IL-18, were increased under *T. pallidum* insult. The expression of active Caspase-1 (C-Caspase-1) with cleavage function and N-GSDMD that forms surface pores also increased. However, MCC950 downgraded the levels of these pyroptosis-related signaling molecules.

The IHC staining indicated that *Treponema pallidum* was located in the seminiferous tubules and the interstitial area. In contrast to our results, Lukehart et al. [20] and Lauderdale et al. [21] found *T. pallidum* only in the interstitial region of the rabbit testis, but not in the seminiferous tubules. The fact that *T. pallidum* is also present in the semen of patients with syphilis [19] also suggests that our results are reasonable. *T. pallidum* invaded the seminiferous tubules of the rabbit testis, which provided the opportunity for direct contact and damage to sperm. Additionally, the H&E staining suggested that the interstitial fibrosis, the seminiferous tubules were significantly atrophied, and the number of mature sperm was remarkably reduced. This finding was consistent with previous studies [19, 20]. Assessment of pyroptosis was conducted by measuring caspase-1 activity and performing Calcein-AM/PI staining [14]. Moreover, the LDH release and cell viability were detected. After stimulation of GC-2spd cells with *T. pallidum*, it was indeed possible to observe an increase in pyroptotic cell death. These results can be alleviated by the NLRP3 inflammasome inhibitor MCC950, indicating that the activation of the NLRP3 inflammasome serves as a key driver for the induction of pyroptosis of GC-2spd cells caused by *T. pallidum*. The NLRP3 expression levels in the *T. pallidum* infection rabbits showed a significant trend in elevation of the testis [10], which is consistent with our data. Furthermore, our in vivo and in vitro data revealed an increase in the expression of pyroptosis-related molecules (ASC, Caspase-1, C-Caspase-1, GSDMD, N-GSDMD, IL-1β, and IL-18). MCC950 acts as a potent and selective small-molecule inhibitor of the NLRP3 inflammasome [22–24]. Administration of MCC950 reduced the expression of pyroptosis-related molecules in GC-2spd cells stimulated by *T. pallidum*, which suggests that inhibition of the NLRP3 inflammasome activation is closely related to a reduction in sperm damage.

The pyroptosis of spermatocytes is not an isolated cellular event. Its clinical consequences directly point to male infertility and permanent damage to testicular function. Spermatocytes are the core stage of sperm production. If a large-scale pyroptosis occurs in spermatocytes, the “assembly line” for sperm production will be directly disrupted. This directly leads to oligospermia (reduced sperm production) or even azoospermia. At the same time, the inflammatory oxidative stress caused by pyroptosis in the testicular microenvironment will also lead to decreased vitality (asthenozoospermia) and abnormal morphology (morphologically abnormal sperm) of the remaining sperm. The continuous and intense inflammatory response (caused by IL-1β, IL-18, etc.) will activate fibroblasts and damage the fine tubule structure within the testis, ultimately resulting in fibrosis and atrophy of the testicular tissue. Once fibrosis occurs, the spermatogenic epithelium will be unable to regenerate, and this damage is irreversible. When spermatocytes undergo pyroptosis, a large amount of their internal “private” antigens will be released. Under healthy conditions, these antigens are isolated from the immune system due to the presence of the blood-testis barrier. Pyroptosis breaks this immune exemption. These exposed antigens will activate the body to produce anti-sperm antibodies. Even if the infection is cured, this autoimmune problem may persist and cause permanent infertility. These explanations account for why some male patients with syphilis (especially those with advanced stages who have not received timely treatment) encounter reproductive difficulties, and provide a strong theoretical basis for clinical doctors to attach great importance to their reproductive health and conduct early intervention and fertility preservation counseling when treating syphilis patients.

Although we have demonstrated that the NLRP3 inflammasome activation and elevated pyroptosis-related molecules under *T. pallidum* insult induced pathological injury of the testis and sperm, our study has some limitations. Since it is impossible to conduct relevant research on patients with syphilis, further studies are also needed to investigate whether male syphilis patients will experience a reduction in the number of sperm cells after being infected with the *T. pallidum*, thereby affecting their fertility within a certain period. In this study, *Treponema pallidum* was directly injected into the testicles. This method is significantly different from the spread of Treponema pallidum throughout the body’s tissues and organs via the bloodstream or lymphatic system upon infection. Therefore, this method may overestimate the extent of testicular damage. In addition, a dual-signal process governs the activation of the NLRP3 inflammasome. The priming signal, mediated through NF-κB activation downstream of Toll-like receptors, ensures the adequate expression of NLRP3 components [25–27]. The subsequent activation signal, which can be instigated by a broad set of cellular disturbances like pore-forming toxins, crystalline substances, potassium efflux, or ROS, then catalyzes the inflammasome complex formation [26, 27]. NLRP3 activation initiates the assembly of a multiprotein inflammasome complex by recruiting downstream adaptor proteins. This complex then activates inflammatory caspases, which subsequently cleave precursor cytokines into their mature forms and execute pyroptosis, a highly inflammatory type of programmed cell death [28–31]. Thus, the lipoproteins on the surface of *Treponema pallidum* may act as PAMPs, upregulating the expression of NLRP3 through the NF-κB pathway (preparation stage). At the same time, *Treponema pallidum* may directly or indirectly (for example, by destroying lysosomes) cause potassium ion efflux from the cells, inducing the massive production of mitochondrial ROS. Subsequently, the combined action of potassium ion efflux and ROS and other signals triggers the assembly of the NLRP3 inflammasome, activates Caspase-1, cleaves and activates IL-1β, IL-18, and Gasdermin D, causing sperm cell pyroptosis. However, further investigation is needed on the upstream signaling pathways that trigger pyroptosis in the pathological damage of the testis and sperm through the NLRP3 inflammasome.

## Conclusions

**Fig. 5:**
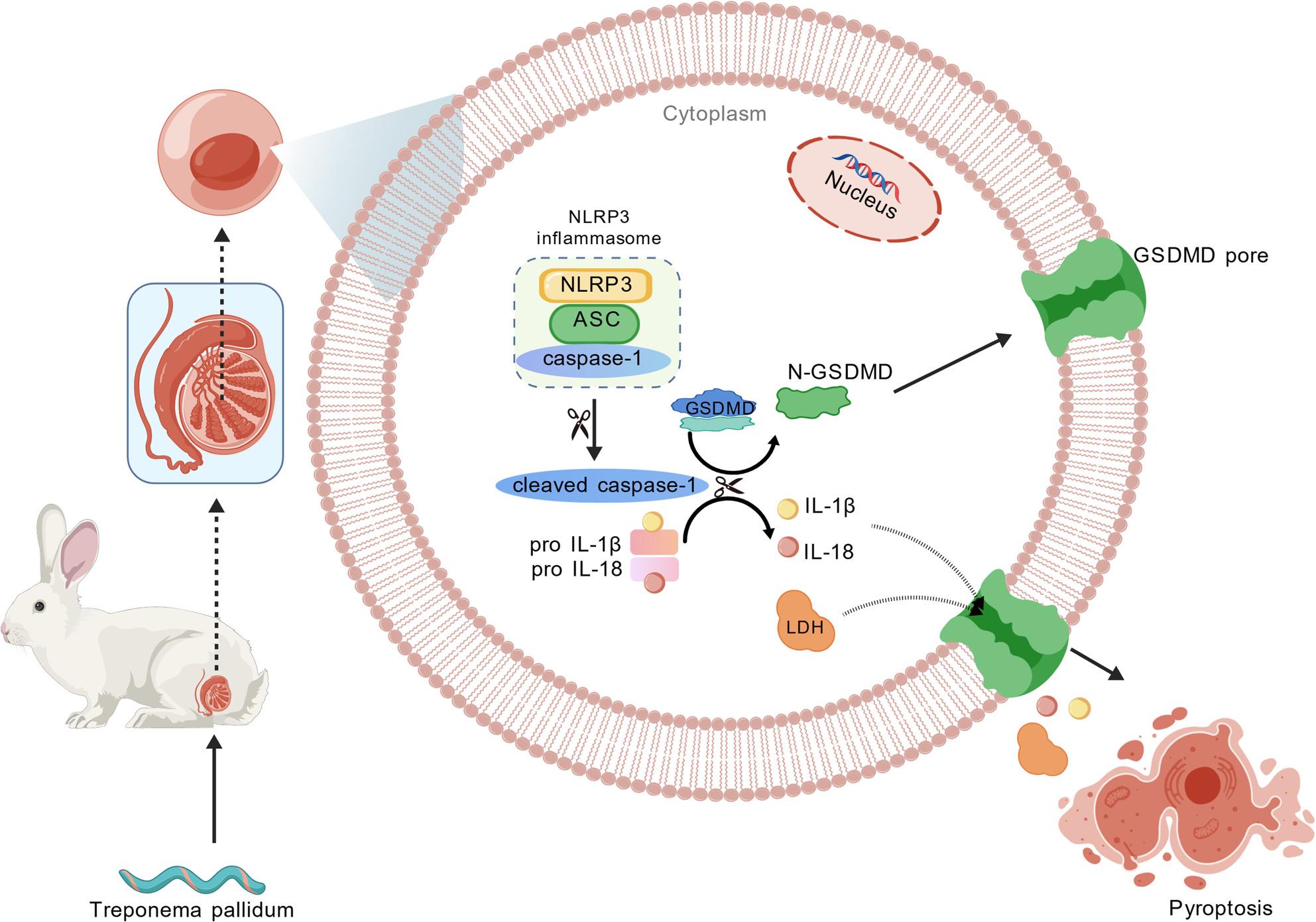
Underlying mechanism of pyroptosis via *T. pallidum* inducing NLRP3 inflammasome activation signaling pathways. Created with BioGDP.com

In summary, this study provides evidence that NLRP3 inflammasome-driven pyroptosis is a key mechanism underlying the testicular and sperm injury observed following *T. pallidum* infection (Fig. 5), and a selective NLRP3 inflammasome inhibitor, MCC950, can attenuate sperm pyroptosis. These results have laid a preliminary experimental foundation for exploring potential intervention strategies, but their clinical significance and application prospects still need to be clarified.

## Supplementary Information

Supplementary Material 1.

## Data Availability

No datasets were generated or analysed during the current study.

## Abbreviations

NLRP3: NOD-like receptor family protein 3
ASC: apoptosis - associated speck - like protein containing a CARD
GSDMD: pore-forming protein gasdermin D
N-GSDMD: GSDMD-N-terminal domain
IL-1β: interleukin-1β
IL-18: Interleukin-18
LPS: Lipopolysaccharide
TPPA: *Treponema pallidum* particle agglutination assay
TRUST: Syphilis Tolulized Red Unheated Serum Test
