# Supplementary figures and images for: Treponema pallidum induces pathological injury of the rabbit testis and sperm through NLRP3 inflammasome activation-mediated pyroptosis
Source: BMC Immunol. 2026 May 9;27:59. doi: 10.1186/s12865-026-00835-7 (PMC13366752; doi:10.1186/s12865-026-00835-7)

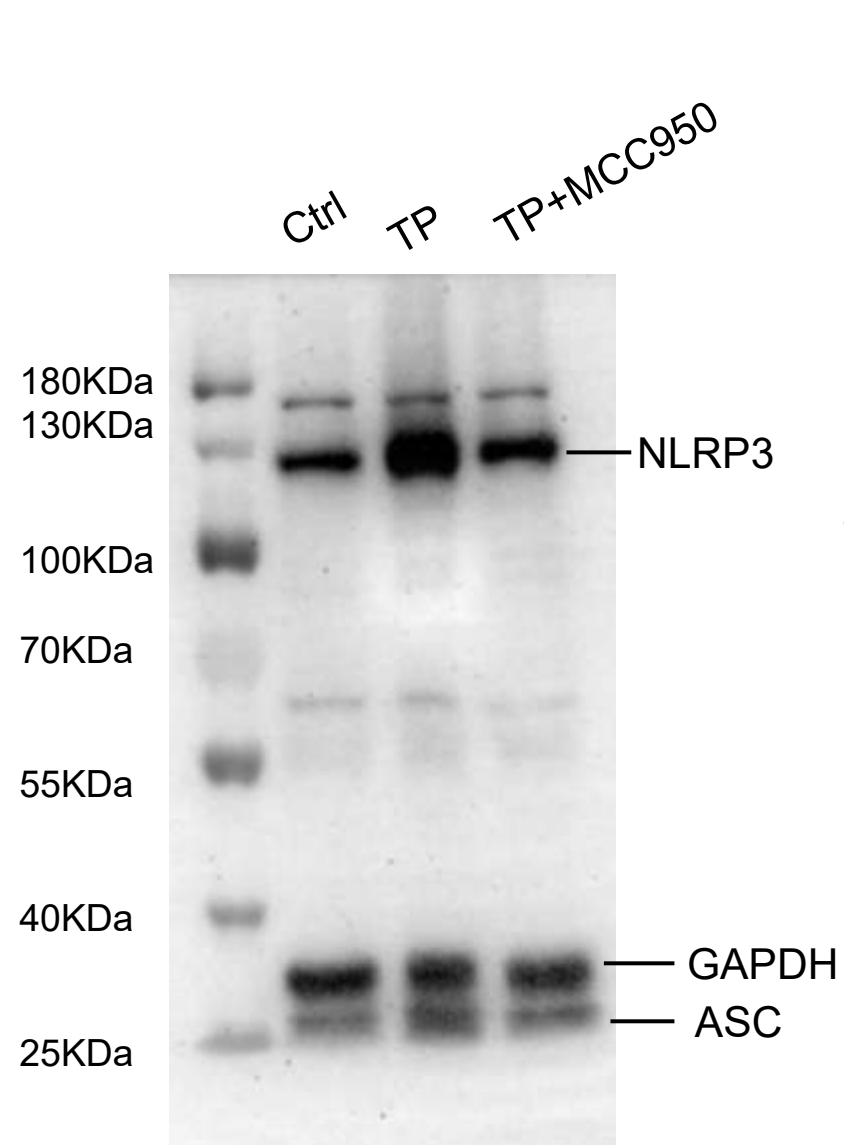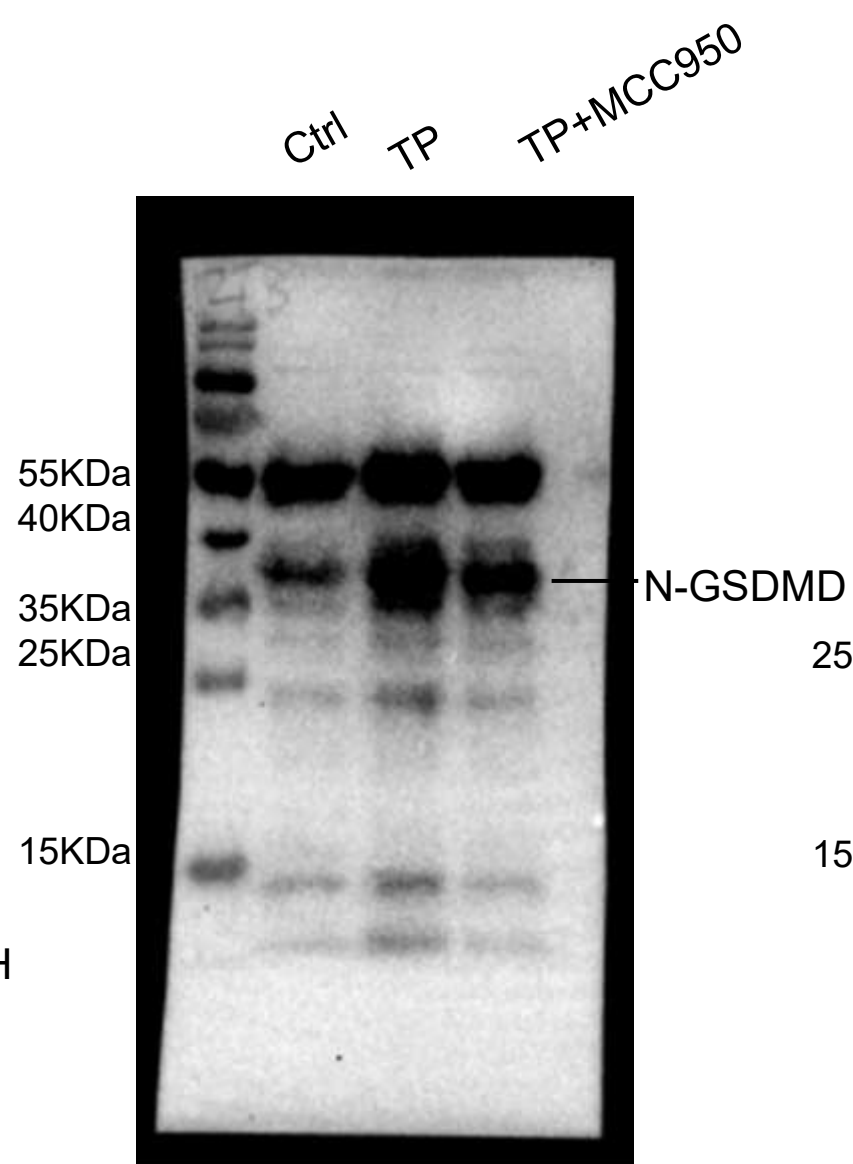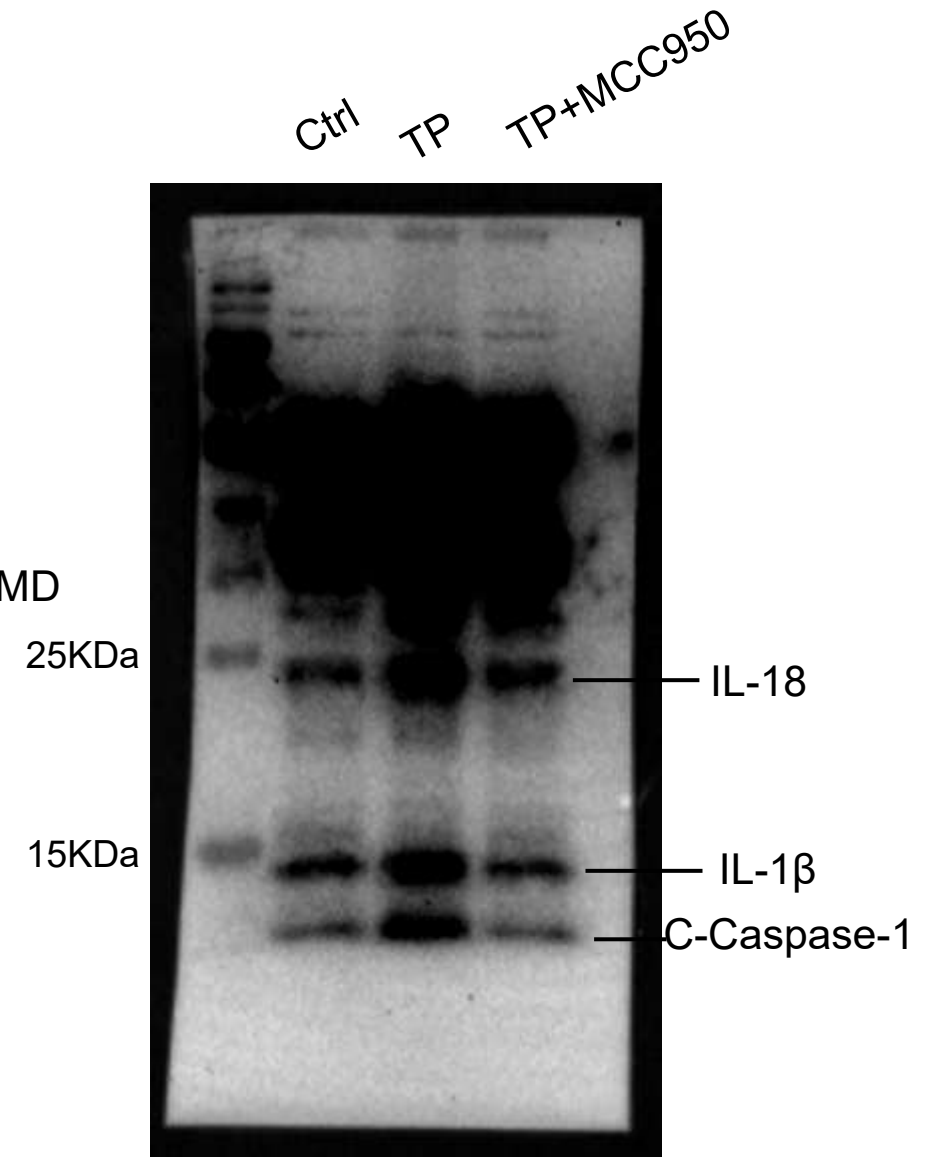

Supplement: Supplementary file 1 — Supplementary Material 1. [file 12865_2026_835_MOESM1_ESM.pdf]
